# Diethyl 2,5-diphenyl­furan-3,4-dicarboxyl­ate

**DOI:** 10.1107/S1600536810045241

**Published:** 2010-11-10

**Authors:** Sheng-Li Hu, Yi-Cheng Le, Ling Huang

**Affiliations:** a Department of Chemistry & Environmental Engineering, Hubei Normal University, Huangshi 435002, People’s Republic of China; b 712th Research Institute, CSIC, Wuhan 430064, People’s Republic of China

## Abstract

In the title compound, C_22_H_20_O_5_, the substituted benzene rings are twisted away from the furan ring, making dihedral angles of 54.91 (14) and 20.96 (15)° with the furan ring. The dihedral angle between the two benzene rings is 46.89 (13)°. One ethyl group of one eth­oxy­carbonyl unit is disordered over two sets of sites with occupancies of 0.56 (12) and 0.44 (12). In the crystal, weak intra­molecular C—H⋯O hydrogen bonds link the mol­ecules into chains along the *c* axis.

## Related literature

For background to the applications of furan-3,4-dicarb­oxy­lic acid and its esters, see: Deshpande *et al.* (2002 ▶). For related structures, see: Hu & Wu (2005 ▶) Hu *et al.* (2005 ▶). For the synthesis, see: Wu *et al.* (1997 ▶).
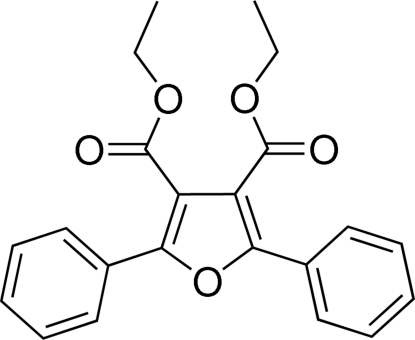


## Experimental

### 

#### Crystal data

C_22_H_20_O_5_
*M* *_r_* = 364.38
Orthorhombic, 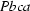

*a* = 11.9535 (8) Å
*b* = 17.0116 (12) Å
*c* = 18.9219 (14) Å
*V* = 3847.7 (5) Å^3^
*Z* = 8
Mo *K*α radiation
μ = 0.09 mm^−1^
*T* = 293 K
0.40 × 0.10 × 0.08 mm

#### Data collection

Bruker SMART APEX CCD area-detector diffractometer
Absorption correction: multi-scan (*SADABS*; Sheldrick, 1997 ▶) *T*_min_ = 0.965, *T*_max_ = 0.993
19957 measured reflections
3778 independent reflections
1952 reflections with *I* > 2σ(*I*)
*R*_int_ = 0.081

#### Refinement

*R*[*F* ^2^ > 2σ(*F* ^2^)] = 0.058
*wR*(*F* ^2^) = 0.160
*S* = 1.01
3778 reflections
268 parameters
6 restraints
H-atom parameters constrained
Δρ_max_ = 0.25 e Å^−3^
Δρ_min_ = −0.27 e Å^−3^

### 

Data collection: *SMART* (Bruker, 2000 ▶); cell refinement: *SAINT* (Bruker, 2000 ▶); data reduction: *SAINT*; program(s) used to solve structure: *SHELXS97* (Sheldrick, 2008 ▶); program(s) used to refine structure: *SHELXL97* (Sheldrick, 2008 ▶); molecular graphics: *SHELXTL* (Sheldrick, 2008 ▶); software used to prepare material for publication: *SHELXTL*.

## Supplementary Material

Crystal structure: contains datablocks global, I. DOI: 10.1107/S1600536810045241/sj5048sup1.cif

Structure factors: contains datablocks I. DOI: 10.1107/S1600536810045241/sj5048Isup2.hkl

Additional supplementary materials:  crystallographic information; 3D view; checkCIF report

## Figures and Tables

**Table 1:** Hydrogen-bond geometry (Å, °)

*D*—H⋯*A*	*D*—H	H⋯*A*	*D*⋯*A*	*D*—H⋯*A*
C4—H4⋯O3	0.93	2.56	3.453 (4)	161

**Fig. 1.**
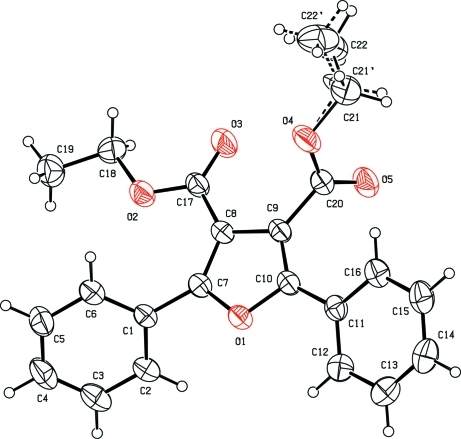
View of the molecule of (I), showing the atom-labelling scheme. Displacement ellipsoids are drawn at the 50% probability level.

**Fig. 2.**
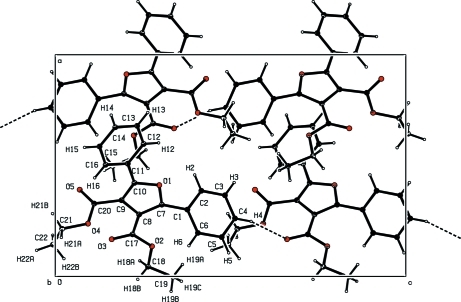
The molecular packing of (I), viewed along the a axis.

## supplementary crystallographic information

### Comment

Furan-3,4-dicarboxylic acid and its esters have been used as starting materials in the synthesis of several bioactive natural products and several pharmacologically useful molecules, preparation of complexes with rare earth metal ions and also as a potential dienes in Diels-Alder reactions for the synthesis of several novel heterocycles (Deshpande *et al.*, 2002). The crystal structures of some furan-3,4-dicarboxylic acid diethyl esters have been reported previously (Hu & Wu,  2005; Hu *et al.*,  2005). In this paper, we report the crystal structure of the title compound, (I). In compound (I), the C1–C6 and C11–C16 phenyl rings form dihedral angles of 54.91 (14) and 20.96 (15)°, respectively with the furan ring, Fig 1. The dihedral angle between the two benzene rings is 46.89 (13)°. In the crystal structure, weak intramolecular C4—H4···O3 hydrogen bonds link the molecules into chains along the *c* axis (Table 1 and Fig. 2).

### Experimental

Compound (I) was synthesized according to the literature procedure (Wu *et al.*, 1997). Single crystals of (I) suitable for X-ray diffraction were obtained by slow evaporation of a methanol solution at room temperature.

### Refinement

The C21/C22 ethyl group of an ethyl carboxylate unit is disordered over two positions with occupancies 0.56 (12)/0.44 (12). Suitable restraints were applied to the O—C and C—C distances involving the disordered atoms. The H atoms were placed in idealized positions and constrained to ride on their parent atoms,with C—H distances in the range 0.97–0.97 Å, and with *U*_iso_(H) =1.5*U*_eq_(C) for methyl H atoms and 1.2*U*_eq_(C) for others. Each methyl group was allowed to rotate freely about its C—C bond.

### Crystal data

**Table:**

C_22_H_20_O_5_	*F*(000) = 1536
*M**_r_* = 364.38	*D*_x_ = 1.258 Mg m^−^^3^
Orthorhombic, *P**b**c**a*	Mo *K*α radiation, λ = 0.71073 Å
Hall symbol: -P 2ac 2ab	Cell parameters from 1354 reflections
*a* = 11.9535 (8) Å	θ = 2.3–16.5°
*b* = 17.0116 (12) Å	µ = 0.09 mm^−^^1^
*c* = 18.9219 (14) Å	*T* = 293 K
*V* = 3847.7 (5) Å^3^	Block, colorless
*Z* = 8	0.40 × 0.10 × 0.08 mm

### Data collection

**Table:**

Bruker SMART APEX CCD area-detector diffractometer	3778 independent reflections
Radiation source: fine-focus sealed tube	1952 reflections with *I* > 2σ(*I*)
graphite	*R*_int_ = 0.081
φ and ω scans	θ_max_ = 26.0°, θ_min_ = 2.2°
Absorption correction: multi-scan (*SADABS*; Sheldrick, 1997)	*h* = −14→14
*T*_min_ = 0.965, *T*_max_ = 0.993	*k* = −18→20
19957 measured reflections	*l* = −23→17

### Refinement

**Table:**

Refinement on *F*^2^	Secondary atom site location: difference Fourier map
Least-squares matrix: full	Hydrogen site location: inferred from neighbouring sites
*R*[*F*^2^ > 2σ(*F*^2^)] = 0.058	H-atom parameters constrained
*wR*(*F*^2^) = 0.160	*w* = 1/[σ^2^(*F*_o_^2^) + (0.0624*P*)^2^ + 0.4808*P*] where *P* = (*F*_o_^2^ + 2*F*_c_^2^)/3
*S* = 1.01	(Δ/σ)_max_ = 0.001
3778 reflections	Δρ_max_ = 0.25 e Å^−^^3^
268 parameters	Δρ_min_ = −0.27 e Å^−^^3^
6 restraints	Extinction correction: *SHELXTL* (Sheldrick, 2008), Fc^*^=kFc[1+0.001xFc^2^λ^3^/sin(2θ)]^-1/4^
Primary atom site location: structure-invariant direct methods	Extinction coefficient: 0.0010 (4)

### Special details

**Table:**

Geometry. All e.s.d.'s (except the e.s.d. in the dihedral angle between two l.s. planes) are estimated using the full covariance matrix. The cell e.s.d.'s are taken into account individually in the estimation of e.s.d.'s in distances, angles and torsion angles; correlations between e.s.d.'s in cell parameters are only used when they are defined by crystal symmetry. An approximate (isotropic) treatment of cell e.s.d.'s is used for estimating e.s.d.'s involving l.s. planes.
Refinement. Refinement of *F*^2^ against ALL reflections. The weighted *R*-factor *wR* and goodness of fit *S* are based on *F*^2^, conventional *R*-factors *R* are based on *F*, with *F* set to zero for negative *F*^2^. The threshold expression of *F*^2^ > σ(*F*^2^) is used only for calculating *R*-factors(gt) *etc*. and is not relevant to the choice of reflections for refinement. *R*-factors based on *F*^2^ are statistically about twice as large as those based on *F*, and *R*- factors based on ALL data will be even larger.

### Fractional atomic coordinates and isotropic or equivalent isotropic displacement parameters (Å^2^)

**Table:**

	*x*	*y*	*z*	*U*_iso_*/*U*_eq_	Occ. (<1)
C1	0.8016 (2)	0.14397 (15)	0.62303 (14)	0.0490 (7)	
C2	0.8864 (3)	0.17700 (17)	0.58313 (15)	0.0605 (8)	
H2	0.9579	0.1817	0.6021	0.073*	
C3	0.8659 (4)	0.2028 (2)	0.51602 (17)	0.0791 (10)	
H3	0.9234	0.2252	0.4898	0.095*	
C4	0.7618 (4)	0.1959 (2)	0.48719 (18)	0.0840 (11)	
H4	0.7483	0.2141	0.4417	0.101*	
C5	0.6774 (3)	0.1621 (2)	0.52514 (18)	0.0820 (11)	
H5	0.6067	0.1569	0.5051	0.098*	
C6	0.6963 (3)	0.13579 (18)	0.59302 (16)	0.0665 (9)	
H6	0.6387	0.1126	0.6186	0.080*	
C7	0.8238 (2)	0.11805 (15)	0.69580 (14)	0.0492 (7)	
C8	0.7797 (2)	0.13356 (15)	0.76053 (14)	0.0482 (7)	
C9	0.8438 (2)	0.08957 (16)	0.81084 (14)	0.0502 (7)	
C10	0.9221 (2)	0.04934 (16)	0.77404 (14)	0.0523 (7)	
C11	1.0088 (2)	−0.00776 (16)	0.79305 (15)	0.0527 (7)	
C12	1.0976 (3)	−0.02123 (18)	0.74782 (17)	0.0644 (8)	
H12	1.1025	0.0058	0.7052	0.077*	
C13	1.1795 (3)	−0.0752 (2)	0.7664 (2)	0.0765 (10)	
H13	1.2394	−0.0841	0.7361	0.092*	
C14	1.1728 (3)	−0.1154 (2)	0.8287 (2)	0.0823 (11)	
H14	1.2277	−0.1519	0.8404	0.099*	
C15	1.0860 (3)	−0.10230 (19)	0.87366 (19)	0.0791 (10)	
H15	1.0820	−0.1295	0.9162	0.095*	
C16	1.0039 (3)	−0.04870 (17)	0.85632 (16)	0.0664 (9)	
H16	0.9449	−0.0399	0.8873	0.080*	
C17	0.6899 (2)	0.18876 (18)	0.77946 (16)	0.0570 (8)	
C18	0.5443 (3)	0.27088 (19)	0.73529 (17)	0.0713 (9)	
H18A	0.5707	0.3181	0.7587	0.086*	
H18B	0.4872	0.2468	0.7645	0.086*	
C19	0.4984 (3)	0.2902 (2)	0.66417 (18)	0.0901 (11)	
H19A	0.5539	0.3179	0.6372	0.135*	
H19B	0.4332	0.3227	0.6694	0.135*	
H19C	0.4785	0.2426	0.6401	0.135*	
C20	0.8287 (3)	0.08891 (18)	0.88887 (16)	0.0598 (8)	
O1	0.91184 (16)	0.06707 (10)	0.70331 (9)	0.0542 (5)	
O2	0.63606 (18)	0.21673 (12)	0.72406 (10)	0.0703 (6)	
O3	0.66800 (19)	0.20603 (17)	0.83882 (12)	0.1034 (9)	
O4	0.7383 (2)	0.04851 (17)	0.90669 (11)	0.1010 (9)	
O5	0.89136 (19)	0.11776 (13)	0.93023 (11)	0.0773 (7)	
C22	0.6437 (8)	0.0896 (6)	1.0062 (4)	0.108 (3)	0.56
H22A	0.6858	0.1376	1.0073	0.163*	0.56
H22B	0.6179	0.0774	1.0530	0.163*	0.56
H22C	0.5806	0.0956	0.9753	0.163*	0.56
C21	0.7149 (11)	0.0258 (6)	0.9805 (5)	0.088 (4)	0.56
H21A	0.6762	−0.0243	0.9826	0.106*	0.56
H21B	0.7834	0.0223	1.0078	0.106*	0.56
C21'	0.7110 (15)	0.0659 (11)	0.9813 (5)	0.107 (6)	0.44
H21C	0.7015	0.1220	0.9882	0.128*	0.44
H21D	0.7700	0.0475	1.0123	0.128*	0.44
C22'	0.6045 (8)	0.0235 (7)	0.9961 (5)	0.116 (4)	0.44
H22D	0.5445	0.0479	0.9705	0.173*	0.44
H22E	0.5888	0.0255	1.0458	0.173*	0.44
H22F	0.6115	−0.0304	0.9815	0.173*	0.44

### Atomic displacement parameters (Å^2^)

**Table:**

	*U*^11^	*U*^22^	*U*^33^	*U*^12^	*U*^13^	*U*^23^
C1	0.0605 (18)	0.0467 (16)	0.0398 (16)	0.0013 (14)	0.0019 (14)	−0.0015 (13)
C2	0.072 (2)	0.0598 (19)	0.0498 (19)	−0.0033 (16)	0.0079 (16)	0.0032 (15)
C3	0.106 (3)	0.078 (2)	0.053 (2)	−0.002 (2)	0.013 (2)	0.0133 (17)
C4	0.124 (3)	0.081 (3)	0.047 (2)	0.024 (2)	−0.003 (2)	0.0108 (18)
C5	0.088 (3)	0.105 (3)	0.053 (2)	0.015 (2)	−0.012 (2)	−0.006 (2)
C6	0.068 (2)	0.083 (2)	0.0479 (19)	−0.0009 (17)	0.0025 (16)	−0.0035 (17)
C7	0.0581 (18)	0.0435 (16)	0.0458 (18)	−0.0024 (14)	−0.0003 (14)	0.0012 (13)
C8	0.0561 (18)	0.0496 (17)	0.0389 (16)	−0.0037 (14)	0.0023 (14)	0.0003 (13)
C9	0.0573 (17)	0.0552 (17)	0.0380 (16)	−0.0107 (15)	−0.0010 (14)	0.0001 (13)
C10	0.0598 (19)	0.0561 (19)	0.0411 (17)	−0.0076 (16)	−0.0020 (15)	0.0014 (14)
C11	0.0583 (19)	0.0511 (17)	0.0486 (18)	−0.0080 (15)	−0.0070 (15)	−0.0027 (14)
C12	0.065 (2)	0.059 (2)	0.069 (2)	−0.0058 (17)	−0.0023 (18)	0.0043 (16)
C13	0.063 (2)	0.073 (2)	0.093 (3)	−0.0019 (19)	−0.0025 (19)	−0.004 (2)
C14	0.077 (3)	0.070 (2)	0.100 (3)	0.0083 (19)	−0.024 (2)	0.002 (2)
C15	0.093 (3)	0.075 (2)	0.069 (2)	0.011 (2)	−0.018 (2)	0.0121 (18)
C16	0.078 (2)	0.064 (2)	0.057 (2)	0.0032 (18)	−0.0064 (17)	0.0031 (16)
C17	0.062 (2)	0.070 (2)	0.0397 (18)	−0.0095 (16)	−0.0018 (15)	−0.0039 (15)
C18	0.068 (2)	0.074 (2)	0.072 (2)	0.0157 (18)	0.0038 (18)	−0.0095 (17)
C19	0.099 (3)	0.096 (3)	0.075 (3)	0.025 (2)	−0.011 (2)	0.002 (2)
C20	0.064 (2)	0.070 (2)	0.0456 (19)	−0.0010 (17)	−0.0030 (17)	0.0004 (15)
O1	0.0654 (13)	0.0538 (12)	0.0434 (12)	0.0046 (10)	0.0026 (9)	0.0025 (9)
O2	0.0822 (15)	0.0820 (15)	0.0465 (13)	0.0269 (12)	0.0012 (11)	−0.0039 (11)
O3	0.0985 (18)	0.164 (3)	0.0481 (15)	0.0494 (17)	−0.0063 (13)	−0.0242 (15)
O4	0.0833 (17)	0.174 (3)	0.0456 (14)	−0.0452 (18)	0.0023 (12)	0.0138 (15)
O5	0.0858 (16)	0.0948 (17)	0.0513 (14)	−0.0113 (13)	−0.0116 (12)	−0.0124 (12)
C22	0.122 (8)	0.127 (8)	0.076 (6)	0.025 (6)	0.022 (5)	0.004 (5)
C21	0.081 (7)	0.103 (9)	0.081 (7)	−0.008 (5)	0.003 (5)	0.018 (5)
C21'	0.121 (12)	0.162 (17)	0.036 (6)	−0.034 (12)	0.029 (6)	−0.015 (8)
C22'	0.119 (9)	0.105 (9)	0.123 (9)	−0.006 (7)	0.049 (7)	0.033 (7)

### Geometric parameters (Å, °)

**Table:**

C1—C2	1.384 (4)	C15—C16	1.379 (4)
C1—C6	1.387 (4)	C15—H15	0.9300
C1—C7	1.470 (4)	C16—H16	0.9300
C2—C3	1.366 (4)	C17—O3	1.190 (3)
C2—H2	0.9300	C17—O2	1.319 (3)
C3—C4	1.364 (5)	C18—O2	1.448 (3)
C3—H3	0.9300	C18—C19	1.491 (4)
C4—C5	1.365 (5)	C18—H18A	0.9700
C4—H4	0.9300	C18—H18B	0.9700
C5—C6	1.379 (4)	C19—H19A	0.9600
C5—H5	0.9300	C19—H19B	0.9600
C6—H6	0.9300	C19—H19C	0.9600
C7—C8	1.359 (3)	C20—O5	1.189 (3)
C7—O1	1.371 (3)	C20—O4	1.324 (4)
C8—C9	1.433 (4)	O4—C21	1.475 (8)
C8—C17	1.470 (4)	O4—C21'	1.479 (8)
C9—C10	1.353 (4)	C22—C21	1.462 (9)
C9—C20	1.487 (4)	C22—H22A	0.9600
C10—O1	1.378 (3)	C22—H22B	0.9600
C10—C11	1.465 (4)	C22—H22C	0.9600
C11—C12	1.383 (4)	C21—H21A	0.9700
C11—C16	1.386 (4)	C21—H21B	0.9700
C12—C13	1.387 (4)	C21'—C22'	1.490 (10)
C12—H12	0.9300	C21'—H21C	0.9700
C13—C14	1.365 (5)	C21'—H21D	0.9700
C13—H13	0.9300	C22'—H22D	0.9600
C14—C15	1.360 (5)	C22'—H22E	0.9600
C14—H14	0.9300	C22'—H22F	0.9600
			
C2—C1—C6	118.8 (3)	C15—C16—C11	120.5 (3)
C2—C1—C7	120.0 (3)	C15—C16—H16	119.7
C6—C1—C7	121.2 (3)	C11—C16—H16	119.7
C3—C2—C1	120.4 (3)	O3—C17—O2	123.6 (3)
C3—C2—H2	119.8	O3—C17—C8	123.2 (3)
C1—C2—H2	119.8	O2—C17—C8	113.1 (2)
C4—C3—C2	120.5 (3)	O2—C18—C19	106.7 (3)
C4—C3—H3	119.7	O2—C18—H18A	110.4
C2—C3—H3	119.7	C19—C18—H18A	110.4
C3—C4—C5	120.0 (3)	O2—C18—H18B	110.4
C3—C4—H4	120.0	C19—C18—H18B	110.4
C5—C4—H4	120.0	H18A—C18—H18B	108.6
C4—C5—C6	120.4 (3)	C18—C19—H19A	109.5
C4—C5—H5	119.8	C18—C19—H19B	109.5
C6—C5—H5	119.8	H19A—C19—H19B	109.5
C5—C6—C1	119.8 (3)	C18—C19—H19C	109.5
C5—C6—H6	120.1	H19A—C19—H19C	109.5
C1—C6—H6	120.1	H19B—C19—H19C	109.5
C8—C7—O1	109.1 (2)	O5—C20—O4	124.1 (3)
C8—C7—C1	135.7 (3)	O5—C20—C9	125.0 (3)
O1—C7—C1	115.2 (2)	O4—C20—C9	110.8 (3)
C7—C8—C9	106.8 (2)	C7—O1—C10	107.9 (2)
C7—C8—C17	128.8 (3)	C17—O2—C18	118.8 (2)
C9—C8—C17	124.2 (2)	C20—O4—C21	122.1 (6)
C10—C9—C8	107.0 (2)	C20—O4—C21'	108.6 (5)
C10—C9—C20	126.2 (3)	C21—O4—C21'	26.8 (8)
C8—C9—C20	126.8 (3)	C22—C21—O4	103.4 (7)
C9—C10—O1	109.1 (2)	C22—C21—H21A	111.1
C9—C10—C11	134.3 (3)	O4—C21—H21A	111.1
O1—C10—C11	116.5 (2)	C22—C21—H21B	111.1
C12—C11—C16	118.9 (3)	O4—C21—H21B	111.1
C12—C11—C10	120.1 (3)	H21A—C21—H21B	109.1
C16—C11—C10	121.0 (3)	O4—C21'—C22'	105.7 (8)
C11—C12—C13	119.7 (3)	O4—C21'—H21C	110.6
C11—C12—H12	120.2	C22'—C21'—H21C	110.6
C13—C12—H12	120.2	O4—C21'—H21D	110.6
C14—C13—C12	120.6 (3)	C22'—C21'—H21D	110.6
C14—C13—H13	119.7	H21C—C21'—H21D	108.7
C12—C13—H13	119.7	C21'—C22'—H22D	109.5
C15—C14—C13	120.2 (3)	C21'—C22'—H22E	109.5
C15—C14—H14	119.9	H22D—C22'—H22E	109.5
C13—C14—H14	119.9	C21'—C22'—H22F	109.5
C14—C15—C16	120.1 (3)	H22D—C22'—H22F	109.5
C14—C15—H15	119.9	H22E—C22'—H22F	109.5
C16—C15—H15	119.9		
			
C6—C1—C2—C3	1.6 (4)	C11—C12—C13—C14	0.3 (5)
C7—C1—C2—C3	−178.6 (3)	C12—C13—C14—C15	−0.6 (5)
C1—C2—C3—C4	−0.4 (5)	C13—C14—C15—C16	0.5 (5)
C2—C3—C4—C5	−0.8 (5)	C14—C15—C16—C11	0.0 (5)
C3—C4—C5—C6	0.8 (5)	C12—C11—C16—C15	−0.4 (4)
C4—C5—C6—C1	0.4 (5)	C10—C11—C16—C15	180.0 (3)
C2—C1—C6—C5	−1.5 (4)	C7—C8—C17—O3	−169.1 (3)
C7—C1—C6—C5	178.7 (3)	C9—C8—C17—O3	5.6 (5)
C2—C1—C7—C8	124.2 (4)	C7—C8—C17—O2	11.2 (4)
C6—C1—C7—C8	−56.0 (5)	C9—C8—C17—O2	−174.0 (2)
C2—C1—C7—O1	−53.9 (3)	C10—C9—C20—O5	69.8 (4)
C6—C1—C7—O1	125.9 (3)	C8—C9—C20—O5	−109.1 (4)
O1—C7—C8—C9	−0.1 (3)	C10—C9—C20—O4	−107.2 (3)
C1—C7—C8—C9	−178.2 (3)	C8—C9—C20—O4	73.8 (4)
O1—C7—C8—C17	175.3 (2)	C8—C7—O1—C10	0.9 (3)
C1—C7—C8—C17	−2.8 (5)	C1—C7—O1—C10	179.4 (2)
C7—C8—C9—C10	−0.6 (3)	C9—C10—O1—C7	−1.3 (3)
C17—C8—C9—C10	−176.3 (2)	C11—C10—O1—C7	177.2 (2)
C7—C8—C9—C20	178.5 (3)	O3—C17—O2—C18	−0.6 (5)
C17—C8—C9—C20	2.8 (4)	C8—C17—O2—C18	179.0 (2)
C8—C9—C10—O1	1.2 (3)	C19—C18—O2—C17	−178.4 (3)
C20—C9—C10—O1	−178.0 (3)	O5—C20—O4—C21	−8.8 (6)
C8—C9—C10—C11	−176.9 (3)	C9—C20—O4—C21	168.3 (5)
C20—C9—C10—C11	4.0 (5)	O5—C20—O4—C21'	17.0 (11)
C9—C10—C11—C12	−160.6 (3)	C9—C20—O4—C21'	−166.0 (10)
O1—C10—C11—C12	21.5 (4)	C20—O4—C21—C22	93.6 (10)
C9—C10—C11—C16	19.1 (5)	C21'—O4—C21—C22	27.6 (18)
O1—C10—C11—C16	−158.9 (3)	C20—O4—C21'—C22'	175.9 (12)
C16—C11—C12—C13	0.2 (4)	C21—O4—C21'—C22'	−58.8 (17)
C10—C11—C12—C13	179.8 (3)		

### Hydrogen-bond geometry (Å, °)

**Table:**

*D*—H···*A*	*D*—H	H···*A*	*D*···*A*	*D*—H···*A*
C4—H4···O3^i^	0.93	2.56	3.453 (4)	161

Symmetry codes: (i) .
